# Analysis of the status of treatment of benign thyroid diseases — a public health problem aggravated in the COVID-19 pandemic era^☆^

**DOI:** 10.1016/j.bjorl.2021.08.008

**Published:** 2021-10-23

**Authors:** Giulianno Molina Melo, Antonio José Gonçalves, Fernando Walder, Carolina Ferraz, Murilo Catafesta Neves, Marcio Abrahão, Onivaldo Cervantes

**Affiliations:** a Universidade Federal de São Paulo, Escola Paulista de Medicina (UNIFESP/EPM), Departamento de Otorrinolaringologia e Cirurgia de Cabeça Pescoço, São Paulo, SP, Brazil; b Beneficencia Portuguesa de São Paulo Hospital, Departamento de Cirurgia de Cabeça Pescoço, São Paulo, SP, Brazil; c Sociedade Brasileira de Cirurgia de Cabeça e Pescoço (SBCCP), Departamento de Tireoide, São Paulo, SP, Brazil; d Faculdade de Ciências Médicas da Santa Casa de Misericórdia de São Paulo, Departamento de Cirurgia – Divisão de Cirurgia de Cabeça e Pescoço, São Paulo, SP, Brazil; e Sociedade Brasileira de Cirurgia de Cabeça e Pescoço (SBCCP), São Paulo, SP, Brazil; f Faculdade de Ciências Médicas da Santa Casa de Misericórdia de São Paulo, São Paulo, SP, Brazil

**Keywords:** COVID-19, Thyroid diseases, Endocrine surgical procedures, Public health, Outcome assessment, Health care

## Abstract

**Objective:**

Goiters and benign nodules detected in the thyroid are growing lesions and the COVID-19 pandemic have negatively impacted on their surgical treatment. The appropriate selection of patients to treatment will improve the overall health status. This article review will focus on the impact of the COVID-19 pandemic on treatment of benign conditions of the thyroid gland and their implications.

**Methods:**

This review pointed out the status of the health system in developing country and the problems to treat benign surgical diseases of thyroid. Aspects of epidemiology, incidence, clinical presentation and surgical treatment of goiters, economic and health status impact were cited.

**Results:**

All surgical treatment of goiter and other benign conditions were postponed, forced to redirect, and reschedule all benign surgeries, situation aggravated by poor public management and closure of hospital beds. These conditions have caused deterioration in patients’ physical (decompensated thyroid disease) and mental health status, increasing work disabilities and burdening society by increasing the social and health cost. The overall situation could be catastrophic in emergent countries where this increased disease-related social expenditure on surgical treatment may increase the risk of national impoverishment as increase the treatment cost. Brazilian Society Head and Neck Surgery related some recommendations and new suggestions were made to safely treat these high potential hazard surgical conditions.

**Conclusions:**

Surgeries for goiter and benign thyroid conditions can be performed during the COVID-19 pandemic, following strict safety protocols for the patient and the medical team, reducing the negative economic and on patient health impact.

## Highlights

• The COVID-19 pandemic have negatively impacted the surgical treatment of Goiters.
• The postponed surgical treatment was worsened by closure of hospital beds and bad public management.
• With safety protocols, surgeries for goiter and benign thyroid conditions can still be performed.
• The restart surgeries for goiters will reduce the negative economic and patient health impact.

## Introduction

“Economics is not about things and tangible material objects; it is about men, their meanings and actions” Ludwig Heinrich Edler von Mises (1881–1973).

The COVID-19 pandemic forced the WHO to come up with a recommendation to postpone all elective surgeries worldwide, supported by national and international class societies, impacting the surgical treatment of the benign thyroid conditions, which include cysts, goiters, toxic goiters, adenomas, and thyroiditis.^1^ The real problems resulted from all this necessary postpone has been little evaluated from standard point of care in these cohort of patients.

This article review will focus only on the impact of the COVID-19 pandemic on treatment of benign conditions of the thyroid gland and their implications, as of June 2021, in an emergent country as Brazil. The present text has the objective of informing and raising more important questions, which are the base of a healthy scientific evolution to be later used in favor of patients.

## Contemporary review

### Benign thyroid diseases

More than 90% of the nodules detected in the thyroid are indolent benign lesions, a very common finding in day-to-day clinical practice and only about 4%–7% are actual carcinomas. In 2%–6% of cases, benign lesions are diagnosed by palpation on clinical examination, by ultrasound findings in 19%–35%, and by incidental post-mortem findings in 8%–65%.^2^, ^3^ Most nodules diagnosed today are approximately 1.0 cm in diameter, are either barely palpable or not palpable at all, and are accompanied by an absence of thyroid dysfunction. Based on previous studies, the prevalence of benign thyroid lesions is high at 6.4% in women and 1.5% in men, occurring in 59.2% in a Brazilian population and 15.85 patients/1000 inhabitants in Korea.^4^, ^5^, ^6^, ^7^

Current studies demonstrate that these nodules present slow, progressive growth, limited to approximately 5 mm in 5 years for the main nodule in cases of multinodular disease. The majority of patients are mostly asymptomatic, do not require treatment, and can be simply referred to clinical follow-up to “wait-and-see” policies in the treatment of these benign thyroid nodules, in a either non-pandemic and pandemic situation.^2^

Otherwise, some other studies also report gradual growth of the nodules and noted a progressive increase in gland volume in the form of multiple nodules which are correlated to progressive growing goiter and an increased risk of hyperthyroidism; in which case the benign condition can become symptomatic, also increasing the cardiologic risk due arrhythmias and the overall surgical risk. Particularly, patients with a family history of thyroid nodules and a high dietary intake of iodine were found to be susceptible; these studies concluded that the gradual increase of thyroid function in such cases is directly related to the increase in the goiter volume, a worrying situation nowadays in COVID-19 era.^8^

Regarding the treatment, a study of 488 patients who underwent surgery for goiters over 15 years, about 25% of goiters were classified as large (between 106 and 176 g) and 75% as small (between 18 and 37 g); obesity and black race were found to be risk factors associated with goiter growth.^9^ In another study, patients with goiter had a high risk of having grade-III obesity, with a strong causal link, due to insulin resistance and increased leptin, leading to thyroid dysfunction and stimulation of thyroid parenchyma growth.^10^

The onset of dysphagia, decubitus dyspnea, foreign-body sensation or globus pharyngeus, Pemberton’s sign (a late sign of cervicothoracic goiter with vascular compression), and a multinodular goiter on palpation, whether associated with hyperthyroidism or not, indicates goiter compression of the respiratory-digestive tract. The associated condition of obstructive sleep apnea syndrome and other comorbidities lead to also an increased risk of mortality in these patients.^11^ This requires prompt surgical treatment due to the risk of aspiration with recurrent pneumonia and difficult clinical treatment.

Compressive goiters may lead to difficult orotracheal intubation in the emergency room. The main indications for goiter surgery are compression of the digestive tract, airways, intrathoracic growth, marked growth during the follow-up period, vascular compression, cosmetic deformity, and risk of malignancy.^12^ Although life threatening non-malignant thyroid condition, the clinical situation above has been delayed in COVID-19 pandemic, when only select cases of malignant histology has been operated, with evident negative impact on the overall patient health, as mentioned somewhere below.

Regarding the type of surgery, in a Brazilian study of 1789 patients who underwent goiter surgery, the goiter was found to be benign in 62.4% (n = 1116) of patients undergoing total thyroidectomy and 37.6% (n = 673) of those undergoing partial thyroidectomy. The authors concluded that total thyroidectomy is effective, showing benefit over partial thyroidectomy, with the same rate of complications, i.e., 12.2% of transient hypoparathyroidism, 1.6% of definitive hypoparathyroidism, 1.9% of transient lower laryngeal nerve injury, and 0.35% of definitive lower laryngeal nerve injury.^13^

Thyroidectomy is the formal treatment indication in medium to large goiters, and total thyroidectomy is superior to partial operation. This surgery is considered safe in experienced hands, with low complication rates: less than 1% for definitive dysphonia due to injury of the laryngeal nerve and about 1% for hypoparathyroidism.^14^

Despite the fact that most surgeons: endocrine surgeons, otorhinolaryngologists, head and neck surgeons and general surgeons are familiarized with the neck anatomy, thyroid anatomy and technical principles of the thyroid surgery and believe that the thyroid surgery is a “safe procedure”; in the COVID-19 pandemic this perception were severely impaired, once more patients with “bigger complicated disease” has been submitted to surgery, increasing the overall complication rate, being of relevant concern.

### The COVID-19 pandemic aspects

The World Health Organization (WHO) was notified about the first cases of an atypical pneumonia in Wuhan, China, on December 31, 2019; the new virus was officially named SARS-CoV-2 on February 11, 2020; the disease it caused was named coronavirus disease 2019 (COVID-19) and the WHO declared COVID-19 a pandemic on March 11, 2020.

The virus can spread by direct, indirect, or close contact (up to 1 m), through aerosol or micro-aerosol particles, salivary and respiratory secretions, when talking, coughing, and sneezing. People become infected when the virus comes into contact with the mucous membranes of the mouth, nose, or eyes. According to data from Johns Hopkins University Coronavirus Resource Center, updated in real time, as of July 03, 2021, 183.274.120 people worldwide have been infected with SARS-CoV-2, with overall death of 3.966.575; of whom 18.687.469 people were located in Brazil. The country had recorded 521.952 deaths, with incidence in June/21 of 65.165 cases/day; and daily mortality of 1.879 patients, resulting in a mean mortality rate of 2.46%, ranging from 1.66% in the Federal District (the administrative area of the national capital city, Brasília) to 5.64% in the State of Rio de Janeiro.^15^

As pandemic evolute, appropriate knowledge has been learned and the health safety policies officially implemented in Brazil included social distancing, personal protection equipment (PPE; use of face masks, alcohol-based hand sanitizer, etc.), and vaccination. Hospitals were also forced to redirect and reschedule surgeries. Only time-sensitive surgeries, such as oncological surgeries and cases in which an imminent risk of life remain indicated (urgency or emergency), postponing the surgical thyroid benign conditions as per recommendations published by the Brazilian Society of Head and Neck Surgery (SBCCP) and by other were performed.^15^, ^16^, ^17^, ^18^

Recently, new SARS-CoV2 variants have been detected worldwide, first in Africa (B.1.351) and England (B.1.1.7), and later in Brazil, (strains P1, P2, and B1.1.33, with mutations in the viral genes N501Y and E484K). Variant P1 was dubbed the “Brazilian variant”, and is characterized by a greater capacity for dissemination and contamination, higher mortality index and a higher growth speed in relation to the common SARS-CoV-2 strains. The P1 strain has resulted in a second wave of the pandemic that caused chaos and health system collapse in the Northern Brazilian city of Manaus in January 2021.^19^, ^20^

The direct consequence of these variants is that the effects of the disease are no longer as limited to elderly and vulnerable patients (those with comorbidities) as the original SARS-CoV-2. Now young patients without comorbidities are also becoming rapidly and severely ill, requiring longer hospitalization time, both in normal and ICU beds. Some of these cases result in death, with a mortality rate of almost 80% for patients who required orotracheal intubation and assisted ventilation.

In Brazil, this situation has currently resulted in an unprecedented occupation of hospital beds, including ICU beds, increased consumption of hospital supplies (anesthetics, antibiotics, anticoagulants, corticoids, etc.) by both the public health system (the so-called Brazilian Unified Health System, or SUS in the Portuguese acronym) and the Private Health System (PHS). This collapse situation of increased hospital length stays, more occupied beds, increased consumption of hospital supplies lead to overall delay during indeterminate time of all the surgical benign thyroid conditions in favor only of the cancer patients; data not officially positioned by the federal government but adopted by the federation states, justified in part by the pandemic, but the immense failures of the Brazilian health system, both in SUS and the PHS, are also partly to blame.

A similar hospital bed occupancy situation was observed in the U.S. from November 2020 to early February 2021, but the rates decreased after that period, probably reflecting the initiation of systematic mass vaccination.^15^

The enormous physical and emotional stress of the entire team of health professionals who directly and indirectly care for these cases should also be mentioned, including physicians of various specialties, nurses, nursing assistants, physical therapists, and psychologists.^21^, ^22^, ^23^, ^24^

### The Public and Private Health System aspects

**Figure 1:**
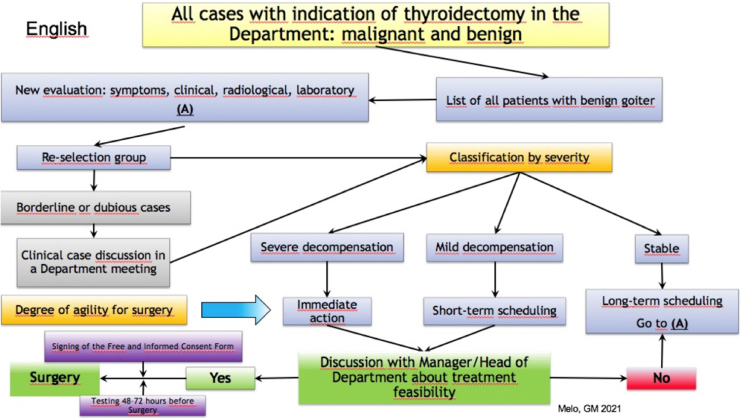
Hospital beds lost in the Brazilian Unified Health System (SUS) and outside SUS in the period from 2008 to 2018.^25^.

In a technical study by the Brazilian National Confederation of Municipalities, it was reported that over a period of 10 years (2008–2018), more than 40,000 hospital beds were lost, with more closures (23,091 beds) than openings (18,000 new PHS beds) in the case of SUS, even before the pandemic. It was also found that the national average is 2.1 beds/1000 inhabitants, which is below the WHO recommendation of 2.5 to 3 beds/1000 inhabitants. Additionally, these beds are unevenly distributed between SUS and the PHS, and also between Brazilian states and municipalities (Fig. 1).^25^

The decrease of hospital beds over the years, the shrinking of the health sector, corruption in the health system, embezzlement of public funds, a decrease in the population’s income, with about 12 million patients migrating from the PHS to SUS, and many other factors all culminated in a health crisis, overwhelming the facilities for patient care in both health systems. This further resulted in a bottleneck at hospitals, reducing vacancies for both hospitalization and surgery.^26^, ^27^, ^28^, ^29^

Another issue is the persistent decrease in reimbursement from SUS to hospitals, up to 77% for the cost of surgeries, a fact that may justify the administrative decisions of managers to reduce admissions to contain the financial crisis in public hospitals.^30^ Another aspect is the difference in medical remuneration paid by SUS when total thyroidectomy is performed for cancer vs for benign goiters, a distortions of the SUS remuneration table that needs to be corrected.^31^ The difference is very large and in favor of oncological cases, even though it is often more laborious to surgically operate on a large goiter than a thyroid cancer.

This difference in remuneration for both medical fees and hospital services makes surgeons and hospitals reluctant to treat patients with benign thyroid diseases. The SBCCP is aware of this issue and has been participating in a group that proposes corrections in the SUS remuneration table, but this is a long and arduous task. Informally, based on unpublished but dynamic data that vary for each head and neck surgery department in Brazil, it has been observed that the duration of care (defined as the time from the first consultation to the hospital discharge after surgery) of patients with benign thyroid diseases (goiter, adenomas) used to vary nationwide from 1 year and 6 months to up to 3 years before pandemic, worsening during COVID-19 era.

As cited, the WHO have come up with a recommendation to postpone all elective surgeries worldwide, supported by national and international class societies; however, head and neck surgery was not included in the initial recommendation.^32^, ^33^ During the COVID-19 pandemic, the safety of the patient and the head and neck surgery team should be paramount, and several guidelines have been posteriorly published in this regard.^17^, ^34^ However, to date, there is no consensus on the best conduct in cases of goiter and benign diseases. However, the results of some guidelines that helped in the selection of patients to be submitted to surgery during the COVID-19 pandemic can be extrapolated.^35^ Other specific guidelines have oriented the ideal moment selection of patient to surgery, taking into account the capacity of the hospital network and the sufficient availability of hospital supplies.^18^

The prioritization of head and neck surgeries during the pandemic was discussed in a Stanford University article suggesting a 30–90 day postponement of goiter surgeries considered less urgent, i.e., in cases with no signs of airway commitment.^36^ It was noted that out of an estimated worldwide volume of 4,845,604 head and neck surgeries scheduled during the pandemic, 3,950,551 (81.5%) were cancelled. In Brazil, almost 247,444 of total surgeries were cancelled in a period of 12 weeks, and no specifically thyroid surgery number were cited; thus, generating a large social impact, which will have a long-term negative effect in health and economic terms, also be harmful for the patients by worsening their disease.^37^

The SBCCP recommendations for the safe resumption of surgical procedures are a landmark in this specialty, by guiding surgeons about surgery indications during the pandemic. The recommendations include the postponement of goiter and benign thyroid surgeries (Item 2), except for “goiters with airway compression and evident respiratory symptoms, and Graves’ disease with contraindications to clinical treatment”.^16^, ^38^

In this respect, the SBCCP took an excellent step by not mitigating the consequences of the postponement of benign thyroid surgeries, understanding the harmful effects of this postponement, and always weighing the risk/benefit ratio during the COVID-19 pandemic. Similar to other manifestations regarding the cancellation or postponement of head and neck surgeries,^39^ the present article aims to alert professionals involved in the treatment of these patients (surgeons, endocrinologists, and multidisciplinary teams) about the risks of further postponing benign thyroid surgeries, even considering all the other associated hindrances mentioned above.

There is a real risk that the postponement of surgery for benign conditions as a result of the pandemic will cause a deterioration in patients’ physical and mental health status, increasing work disabilities and burdening society by increasing the social cost. This could be catastrophic in emergent countries where this increased disease-related social expenditure on surgical treatment may increase the risk of national impoverishment.^37^, ^40^ There are those who argue, not without reason, that the slow growth of goiter and benign thyroid conditions are not an aggravating factor for the patient, and it is possible to wait until safer times. However, this line of argument does not take into consideration the fact that goiters with “borderline” indication for surgery today will, in a short time, either grow, clinically deteriorate or cause compression, thus making the surgical act more exhaustive, more time-consuming, and with a higher risk of complications; almost 35% of growing goiters becoming substernal (grade II–III) will require sternotomy, with high risk to dysphonia (OR = 14.29) and transient hypoparathyroidism (OR = 4.48);^41^, ^42^, ^43^, ^44^, ^45^ the risk of surgical morbidity rate in toxic goiter is nearly 37%^44^ and the mortality can be high as 3.1% in the postoperative period.^43^ For goiters that already have surgical indication due to compressive symptoms or hyperthyroidism, every effort should be made for their prompt surgical resolution, while following the due safety measures mentioned before.

Thus, surgeries can be performed during the pandemic, as long as safety protocols for both the patient and the surgical team are adopted, to decrease the impact of postponing surgeries, both from the patient’s health and from the economic point of view, as reported by other surgical departments, respecting, of course, the main present law stablished at the time.^46^, ^47^

### Proposed suggestions

**Figure 2:**
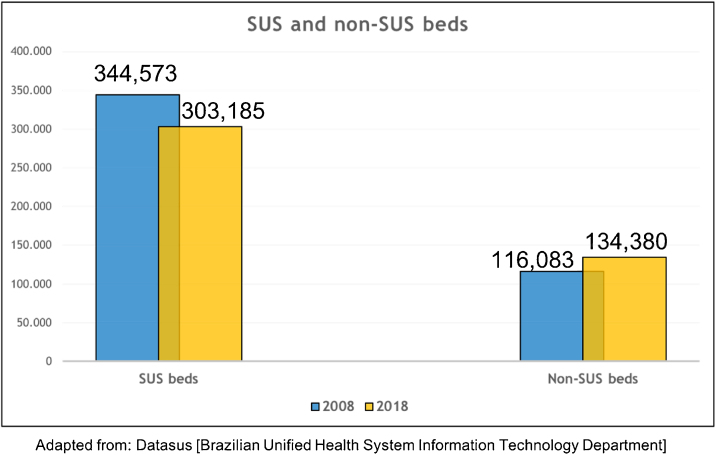
Proposed flowchart for the conduct in surgical cases of goiter and benign thyroid conditions during the COVID-19 pandemic.

We believe the following suggestions can help guide the surgical outcome in cases of goiter and benign thyroid conditions, although they are not intended to provide an extensive coverage of the subject. The authors also present a flowchart with the propositions for better management of goiter, retrosternal goiter, toxic goiter, and benign thyroid cases, classified at the physical and radiologic exam of the thyroid gland (Fig. 2). The goiter were defined as retrosternal according to the Eschapase’s definition (3 cm below the sternal manubrium),^44^ or grade II and III degree of extension according to the cross-section imaging CT system,^42^ with good correlation to intraluminal compression. The severity of clinical decompensation was divided in: Stable – when all clinical and laboratory findings were the same as previous clinical patient data; Mild – when there were laboratory alterations as low TSH with normal Thyroid hormones concentrations or mild symptoms of respiratory discomfort; and Severe – when there were severe clinical signals and symptoms alterations, as weight of loss, palpitations, hypertension, tremor of extremities, fatigue, anxiety, shortness of breath, evident airway and pharynx worsening compression, low TSH and high thyroid hormones concentrations compared to previous exams.

Cases of patients with goiter and benign pathologies should be placed in a separate list of thyroidectomies, excluding carcinomas, to give an estimate of the number of patients.

Once patients are selected, they may be recalled and re-evaluated for symptoms, imaging, and laboratory tests, in an attempt to re-select those with worse symptoms or conditions to receive immediate treatment.

If there is doubt regarding the severity of a case in this new selection, a clinical meeting of the department can be held to discuss the case, similarly to the “tumor boards” for oncologic cases.

Cases should then be reclassified according to severity as either stable, mild decompensation, and severe decompensation. This will allow the medical team to establish the degree of urgency required in the treatment process: immediate action, short-term scheduling, or long-term scheduling.

Once this new list is obtained, the feasibility of treating cases must be jointly discussed with the hospital manager or head of the department, while assessing the current situation of beds, staff, and supplies needed for surgery, as well as the current situation of COVID-19 in that hospital and municipality, aiming at patient and staff safety.

Signature of a free and informed consent form.

Negative COVID19 tests taken 48–72 h before surgery for all patients must be submitted.

Admission to an isolation ward, following the safety standards for COVID-19.

Surgery with minimal aerosols and precautions for the anesthesia and surgical team.

## Conclusion

It can be concluded that surgeries for goiter and benign thyroid conditions can still be performed during the COVID-19 pandemic, as long as safety protocols are followed for the patient and the medical team. This will help in reducing the negative economic impact as well as the impact on patient health.

## Conflicts of interest

The authors declare no conflicts of interest.
